# The Antibody Response Against Neuraminidase in Human Influenza A (H3N2) Virus Infections During 2018/2019 Flu Season: Focusing on the Epitopes of 329-*N*-Glycosylation and E344 in N2

**DOI:** 10.3389/fmicb.2022.845088

**Published:** 2022-03-21

**Authors:** Jing Ge, Xiaojing Lin, Jinlei Guo, Ling Liu, Zi Li, Yu Lan, Liqi Liu, Junfeng Guo, Jian Lu, Weijuan Huang, Li Xin, Dayan Wang, Kun Qin, Cuiling Xu, Jianfang Zhou

**Affiliations:** ^1^Key Laboratory for Medical Virology, National Health, and Family Planning Commission, Chinese Center for Disease Control and Prevention, National Institute for Viral Disease Control and Prevention, Beijing, China; ^2^The Disease Control and Prevention of Qinhuai District, Nanjing, China; ^3^Qinhuai District Center for Disease Control and Prevention, Nanjing, China

**Keywords:** influenza, seasonal H3N2, anti-neuraminidase antibody, antigenic drift, cross-reactivity, immune print

## Abstract

Seasonal influenza A (H3N2) virus has been a concern since its first introduction in humans in 1968. Accumulating antigenic changes in viral hemagglutinin (HA), particularly recent cocirculations of multiple HA genetic clades, allow H3N2 virus evade into humans annually. From 2010, the binding of neuraminidase (NA) to sialic acid made the traditional assay for HA inhibition antibodies (Abs) unsuitable for antigenicity characterization. Here, we investigated the serum anti-NA response in a cohort with a seroconversion of microneutralizing (MN) Abs targeting the circulating strain, A/Singapore/INFIMH-16-0019/2016 (H3N2, 3C.2a1)-like, a virus during 2018/2019 flu seasons. We discovered that MN Ab titers show no difference between children and adults. Nevertheless, higher titers of Abs with NA activity inhibition (NI) activity of 129 and seroconversion rate of 68.42% are presented in children aged 7–17 years (*n* = 19) and 73.47 and 41.17% in adults aged 21–59 years (*n* = 17), respectively. The MN Abs generated in children display direct correlations with HA- and NA-binding Abs or NI Abs. The NI activity exhibited cross-reactivity to N2 of H3N2 viruses of 2007 and 2013, commonly with 329-*N*-glycosylation and E344 in N2, a characteristic of earlier 3C.2a H3N2 virus in 2014. The percentage of such viruses pronouncedly decreased and was even replaced by those dominant H3N2 viruses with E344K and 329 non-glycosylation, which have a significantly low activity to the tested antisera. Our findings suggest that NI assay is a testable assay applied in H3N2 infection in children, and the antigenic drift of current N2 should be considered for vaccine selection.

## Introduction

Influenza is a contagious, acute respiratory disease caused by influenza viruses. Type A influenza virus, including subtypes 1–16 of hemagglutinin (HA) and 1–9 of neuraminidase (NA), has a broad host range and has caused substantial human morbidity and mortality. Seasonal H1- and H3- and N1- and N2-subtype influenza viruses circulate in the human population. Since the first introduction of H3N2 into humans in 1968, its HA gene and antigenicity have undergone extensive changes (Fonville et al., 2014, 2016; Allen and Ross, 2018; Shi et al., 2019). HA Clade 3 H3N2 virus predominated from 2010 and cocirculated with the A(H1N1) pdm09 virus (Falchi et al., 2011). The 3C.2a H3N2 virus emerging at the end of 2013 followed by the 3C.3a in the 2013/2014 flu season has persisted until the present (Lin et al., 2017; Skowronski et al., 2019). There are several explanations for the prevalence of 3C.2a, including its HA gene diversity, the mismatching of vaccine strains A/Texas/50/2012 (3C1, vaccine of 2013/2014 and 2014/15) and A/HongKong/4801/2014 (3C.2a, vaccine of 2016/2017 and 2017/2018), to the circulating strains or the egg-adapted modification of vaccine strain (A/Singapore/INFIMH-16-0019/2016, 3C.2a1, vaccine of 2018/2019) (Chambers et al., 2015; Wu et al., 2017, 2019; Zost et al., 2017; Glatman-Freedman et al., 2020; Skowronski et al., 2020), as well as a possible negative interacting with imprinted immunity in 35–54-year-old adults (Skowronski et al., 2019). Recently, the findings including the N2 antigenicity of 3C.2a1 H3N2 altered by the glycosylation in NA 245 and P468H of 3C.2a H3N2 (Wan et al., 2019), together with non-neutralizing antibodies (Abs) and NA activity inhibition (NI) Abs against contemporary 3C.2a H3N2 viruses in the middle-age group (Gouma et al., 2020), implied the roles of NA.

With the emergence of the variants bearing D151G in N2 (Lin et al., 2010, 2012) in 2010 that confers erythrocytes agglutination with NA, the traditional assay for hemagglutination inhibition (HI) Abs is unsuitable for antigenicity characterization of circulating H3N2 strains. Several new assays for H3N2 antigenicity analysis, such as *h*igh-content *i*maging-based micro-*n*eutralization *t*est (HINT) (Jorquera et al., 2019) and microneutralizing (MN) assay (Lin et al., 2015), have been developed. The anti-HA and anti-N1 Abs induced by virus infection or vaccine (Couch et al., 2013; Cardoso et al., 2014; Changsom et al., 2016) have been comprehensively investigated, while the understanding of anti-N2 is limited. The findings that seasonal H1N1 or H3N2 infection induces a higher frequency of NA-reactive B cells than HA, but rarely in vaccination (Chen et al., 2018), emphasize the data-acquiring need for an anti-N2 response during natural infection.

During the 2018/2019 flu season, we collected the paired sera from 203 children 5–17 years old and 413 adults aged 18–59 years in Northern China (Xu et al., 2021). The current work aimed to explore the anti-N2 responses in the individuals who showed seroconversion of MN Ab titers to the circulating H3N2 strains, indicating being naturally infected. We find the MN Ab titers against the circulating A/Singapore/INFIMH-16-0019/2016-like strain (H3N2, 3C.2a1, SN16/16) show no difference between children and adults, whereas the serum NI Ab titers to SN16/16 N2 and conversion rate in children are higher than that of adults. We demonstrate the correlations of neutralizing Abs with the anti-N2 Abs, including NI- and N2-binding Abs in children. Furthermore, the NI Abs cross-react with N2 of prior seasonal H3N2 viruses of 2007 and 2013, even avian N2 of 1968 H3N2 and 2009 H9N2 viruses. The NI Abs displayed higher activity to the seasonal N2 bearing 329 *N*-glycosylation and E344. Since 2015, the percentage of viruses with such epitopes decreased pronouncedly and was finally replaced by those dominant viruses with glycosylation loss in 329 and E344K substitution in 2019. The NI titer of the antisera to the virus with the alternative epitope was markedly reduced, particularly to 344K.

## Materials and Methods

### Cohort in the Study

The cohort, including 203 children 5–17 years old and 413 adults 18–59 years old, was enrolled in the pre– and post–flu season of 2018/2019. The overall infection incidence based on serological tests was reported in our previous work (Xu et al., 2021). The research protocol was approved by the institutional review board of the National Institute for Viral Disease Control and Prevention, Chinese Centers for Disease Control and Prevention, and written informed consent was obtained from the participants. Among the 203 children 5–17 years old and 413 adults 18 to 59 years old, there were 19 children and 17 adults with a ≥ 4-fold rise in MN Abs against A/Singapore/INFIMH-16-0019/2016(H3N2, SN16/16). The titer of < 10 will be assigned as a value of 5 for statistical purposes.

### Cell Culture

MDCK (Madin–Darby canine kidney)–SIAT1 cells stably transfected with human CMP-N-acetylneuraminate:β-galactose α-2-6-sialyltransferase overexpress the α-2-6–linked sialic acid receptor compared with MDCKs (Hatakeyama et al., 2005). The MDCK-SIAT1 cells are kindly gifted from the US Centers for Disease Control and Prevention. MDCK and human embryonic kidney (293T) cells were obtained from the American Type Culture Collection. The cells were cultured in Dulbecco modified eagle medium (Invitrogen, Carlsbad, CA, United States) supplemented with 10% fetal bovine serum (Invitrogen) and glutamine (2 mM; Invitrogen).

### Generation of Reassortant Viruses

The influenza virus gene segments were amplified by reverse transcription–polymerase chain reaction and cloned into a modified version of the bidirectional expression plasmid pHW2000. The reassortant H6N2 viruses bear the HA of A/Taiwan/1/2013(H6N1) and NAs of the tested viruses, including SN16/16(H3N2, 3C.2a1), A/Hongkong/2671/2019 (H3N2, 3C.2a1b1b, HK2671/19), A/Kansas/14/2017(H3N2, 3C.3a, KS14/17), A/Brisbane/10/2007(H3N2, clade2, Br10/07), A/Switzerland/9715293/2013(H3N2, 3C.3a, SWZ/13), A/Hongkong/1968(H3N2, HK/68), or A/Hongkong/33982/2009(H9N2, HK/09), respectively, and the internal genes of A/Puerto Rico/8/1934(H1N1) (PR8). The H6N2 viruses were rescued by the reverse-genetic (RG) technology with the cotransfection of eight bidirectional plasmids. The viruses were propagated in 9–11-day-old embryonated chicken eggs and stored at −80°C until use. The single or two mutations, N329T, E344K, or T329N, K344E, were introduced into the corresponding pHW2000 plasmid containing the NA segment using appropriate primers and QuikChange™ Site-Directed Mutagenesis Kit (Stratagene, La Jolla, CA, United States), following the manufacturer’s instructions. All plasmids were sequenced to ensure the presence of the introduced mutations and the absence of undesired mutations.

### Microneutralizing Assay

One hundred TCID50 SN16/16 H3N2 virus was precultured with serially diluted sera for 1 h at 37°C and then transferred to the MDCK-SIAT1 monolayer cells in 96-well plates for 18 h at 37°C in a 5% CO_2_ incubator. As reported previously, the cells were processed to be stained by anti-NP (Millipore, Burlington, MA, United States) (Rowe et al., 1999). The IC_50_ of the tested sera was defined as the reciprocal of the last dilution that caused a 50% reduction of OD compared with the virus control.

### Neuraminidase Activity Inhibition by Enzyme-Linked Lectin Assay

As previously reported (Eichelberger et al., 2016; Yu et al., 2017), the tested RG H6N2 viruses were on fetuin-coated 96-well plates for 18 h at 37°C. After the plate was washed, horseradish peroxidase (HRP)–conjugated peanut agglutinin (Sigma–Aldrich, St. Louis, MO, United States) was added. The galactose was exposed by removing the sialic acids, and the fetuin plate was detected by tetramethylbenzidine and measured at 450 nm. The maximum signal within the linear range of the titration curve was selected. The heat-treated sera were serially diluted and then incubated with the tested RG viruses and were performed as the above steps. The IC_50_ of the serum was analyzed by using GraphPad Prism software, version 5.

### Hemagglutinin - and Neuraminidase-Binding Enzyme-Linked Immunosorbent Assay

Recombinant H3 of SN16/16 and N2 of A/Babol/36/05(H3N2, Babol36/05) and KS14/17 were purchased from Sino Biological Inc., China. The 96-well plates were coated with 50 ng per well of the proteins and incubated with serially diluted sera. The bounded Abs were detected using the rabbit anti-human immunoglobulin G–HRP (Sino Biological Inc.), developed by tetramethylbenzidine, and stopped by 2 M H_2_SO_4_, and OD was measured at 450 nm. The titer of the tested sera was defined as the reciprocal of last dilution with an OD value of threefold compared with the blank control.

### The Analysis of Amino Acids at N2 329 and 344 Sites

The HA and NA gene sequences of the H3N2 viruses from January 2014 to December 2021 in the Global Initiative on Sharing All Influenza Data (GISAID) database were downloaded. The Bioedit 7 program was used to align and analyze amino acid residues. The clade was classified as HA clade.

### Statistical Analysis

All graphing and statistical analyses were carried out using R software (version 4.1.2)^1^ and GraphPad Prism software (version 5). The Ab titer and the fold-increase values were used for the response descriptions. Data are expressed as mean ± 95% confidence interval (CI). Mann–Whitney *U*-test was used for comparing differences between the values of children and adult groups. Wilcoxon signed rank test was used for the paired data of children or adult group. Spearman rank correlation analysis was performed on the fold increase of Ab titers. The probability *p* < 0.05 was considered to indicate statistical significance and marked with one asterisk; *p* < 0.01 was marked with two asterisks.

## Results

### Microneutralizing Ab Response in the Studied Population

**FIGURE 1:**
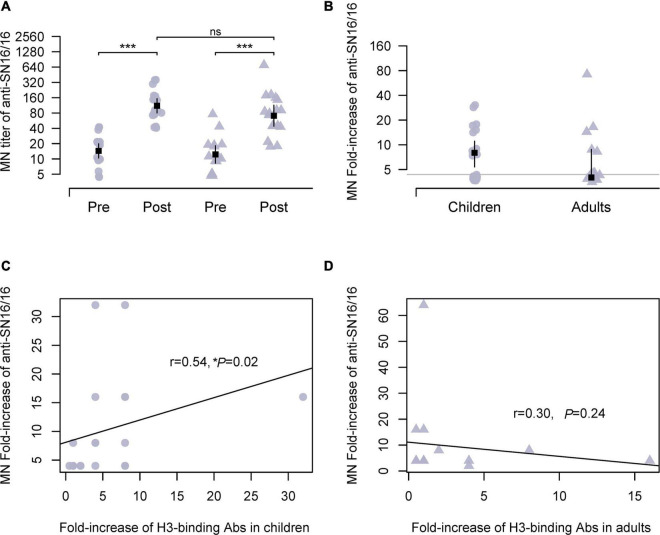
MN Abs against circulating H3N2 virus during 2018/2019 flu season in studied children and adults and their correlations with specific HA-binding Abs. **(A)** Ab titer of MN Abs in the paired sera against the vaccine strain A/Singapore/INFIMH-16-0019/2016(H3N2, SN16/16). **(B)** Fold increase of MN Abs in paired sera against SN16/16; the gray line is the level of 4, positive conversion is fold increase ≥ 4. **(C,D)** Correlations between MN Abs seroconversion and SN16/16 H3-binding Abs in children and adults. **(C,D)** Are the regression line. The dot denotes children’s case, and the triangle represents adults. The mean ± 95% CI is shown. The *r*- and *p*-values are indicated. * < 0.05, *** < 0.001. ns denotes no significantly different.

We performed a seroepidemiological investigation on the paired sera using the vaccine strains (Supplementary Table 1). The traditional HI is for H1N1 and type B influenza virus. Besides the data obtained by optimized HI in the presence of oseltamivir carboxylate, we also used MN by anti-NP staining for the H3N2 virus. The assay was based on the infection and 1–2-life-cycle viral replication in which the surface glycoproteins, HA and NA, and viral RNP complex were all involved. The numbers of MN seroconverts were 17 adults (21–59 years old) and 19 children (7–17 years old), respectively. The Ab titer and fold increase of postseason sera showed no difference (136.8 vs. 111.8; 10.32 vs. 9.30) between children and adults (Figures 1A,B). As some seroconverts against SN16/16 were also seroconverting to type B or A(H1N1) pdm09, we assayed the specific HA-recognizing of the antisera. A significant correlation between the MN Abs and H3-binding Abs was found in children (*r* = 0.54, *p = 0.02; Figure 1C). In contrast, no correlation was found in adults (*r* = 0.30, *p* = 0.24; Figure 1D). Those coincident data support the serodiagnosis for H3N2 infection in children cases.

### Stronger NA Activity Inhibition Ab Response in Children

**FIGURE 2:**
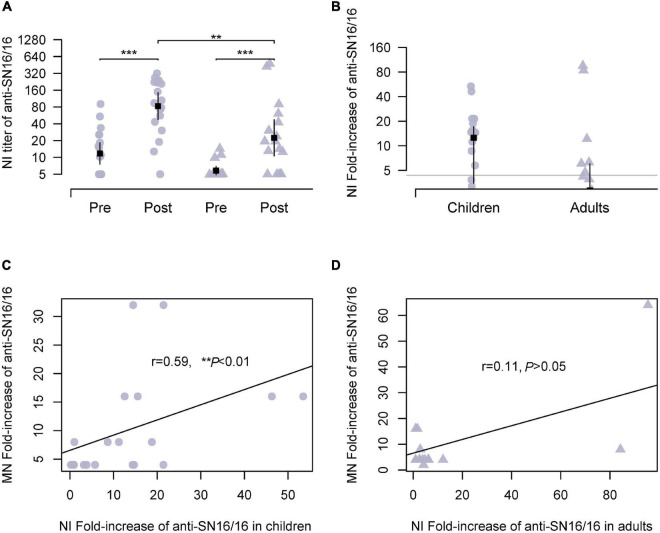
NI Abs against circulating H3N2 virus during 2018/2019 flu season in studied children and adults and their correlations with MN Abs. Recombinant H6N2(RG) virus bearing N2 of SN16/16 was used for the NI test. **(A)** Ab titer of NI Abs in the paired sera against the RGH6N2-SN16/16. **(B)** Fold increase of NI Abs in paired sera against RGH6N2-SN16/16; the gray line is the level of 4; positive conversion is fold increase ≥ 4. **(C,D)** Correlations between MN Abs seroconversion and anti–RGH6N2-SN16/16 NI Abs in children and adults. **(C,D)** Are the regression line. The dot denotes children’s case, and the triangle represents adults. The mean ± 95% CI is shown. The *r*- and *p*-values are indicated. ** < 0.01, *** < 0.001.

The NA of influenza virus cleaved sialic acid linkage and aided virus release during infection. Moreover, NA was another important antigen for host immunity. We expected the possible use of anti-NA Abs for the serological tests of the H3N2 virus other than the modified HI assay. Because of the steric interrupting of anti-HA Abs (Chen et al., 2019; Kosik et al., 2019), a panel of recombinant H6N2 viruses bearing the HA of group 1 HA and the tested N2 was used in our experiments. As expected, NI seroconversion targeting SN16/16 N2 was detected in both adults and children infection cases. In children, the Ab titer of NI was approximately 129 and was significantly higher than in adults (73.47, **p < 0.01; Figure 2A). The conversion rates in children were 68.42 and 41.17% in adults (Figure 2B). To explore the role of NI Abs, we analyzed the correlations between MN Abs and NI. A significant correlation was found in children (*r* = 0.59, **p < 0.01; Figure 2C), not in adults (*r* = 0.11, *p* > 0.05; Figure 2D). A possible role of protecting NI Abs in children suggested that NI assay could be applied to diagnose H3N2 infection and functional evaluation in children.

### Cross-Reactivities of Anti-N2 Abs

We compared the phylogenetic tree of N2 (Supplementary Figure 1). Supplementary Figure 1 (the phylogenetic tree) suggests that N2 subtype influenza viruses could be classified into two prominent clades, corresponding to avian and mammalian (human/swine) influenza viruses. The phylogenetic relationships were largely consistent with the previous study (Fanning et al., 2000). The genetic distance of N2 of 1999–2004 showed a minor difference (Sandbulte et al., 2011). Clade 3 H3N2 viruses have been prevalent since 2010 and cocirculated with (H1N1) pdm09 (Falchi et al., 2011). The clade 3C.2a was dominant and chronologically evolved into 3C.2a1b2a of 2021. Then, we compared the epitopes of the NAs from 1968 to 2021 (Supplementary Table 2). The antigenic epitopes at the head of N2, including D’197–199, F’329–339, G’344–347, I’368–369, and K’400–403, as well as the regions of E’302–308, H’356–358, J’384–386, and L’463–468, were analyzed. Several amino acid substitutions were found in the earlier 3C.2a H3N2 virus from those circulated subsequently. A striking difference was found in N2 of SN16/16 (3C.2a1) and KS14/17 (3C.3a), *N*-glycosylation at 329, and E at 344 of SN16/16 versus non-glycosylation at 329 and K at 344 of KS/14/17. A detailed analysis was performed for the 3C.2a and 3C.3a H3N2 viruses from January 2014 to December 2021 (Table 1). The percentage of viruses with the epitopes of *N*-glycosylation and E344 decreased pronouncedly, although the vaccine strain HK2671/19 of 2020/21 flu season bore non-glycans at 329 and E344 (Supplementary Table 2). Then, the epitopes of glycan loss in 329 and E344K predominated in 3C.2a1b2a, and its representative virus was A/Darwin/9/2021 (H3N2). Unexpectedly, the *N*-glycosylation on 329 in the 3C.2a1b2a H3N2 viruses with K344 reemerged after September 2021, implying those mutations related to viral fitness and evolution in humans.

**TABLE 1:** The amino acid substitutions in NA 329 and 344 of 3C.2a and 3C.3a H3N2 viruses from January 2014 to December 2021.

HA clade	329 N-glycosylation (%)	344 E/K (%)
3C.2a2014 (*n* = 63)	100	E (100)
3C.3a2014 (*n* = 69)	100	E (100)
3C.2a2015 (*n* = 147)	95.2	E (100)
3C.3a2015 (*n* = 36)	72.2	E (100)
3C.2a2016 (*n* = 181)	86.7	E (100)
3C.3a2016 (*n* = 54)	29.6	E (81.5), K (18.5)
3C.2a2017 (*n* = 107)	54.2	E (99.1), K (0.9)
3C.3a2017 (*n* = 28)	67.9	E (35.7), K (64.3)
3C.2a2018 (*n* = 187)	11	E (100)
3C.3a2018 (*n* = 52)	4	K (100)
3C.2a2019 (*n* = 35)	0	E (100)
3C.3a2019 (*n* = 20)	0	K (100)
3C.3a2020 (*n* = 39)	0	K (100)
3C.2a2020 (*n* = 345)	0.6	E (1.2), K (98.8)
3C.3a2021 (*n* = 3)	0	K (100)
3C.2a2021 (*n* = 428)	25.2	E (0.5), K (99.5)

*From January 2014 to December 2021, 1,794 NA sequences were downloaded from GISAID for amino acid substitutions at 329 and 344 analyses.*

**FIGURE 3:**
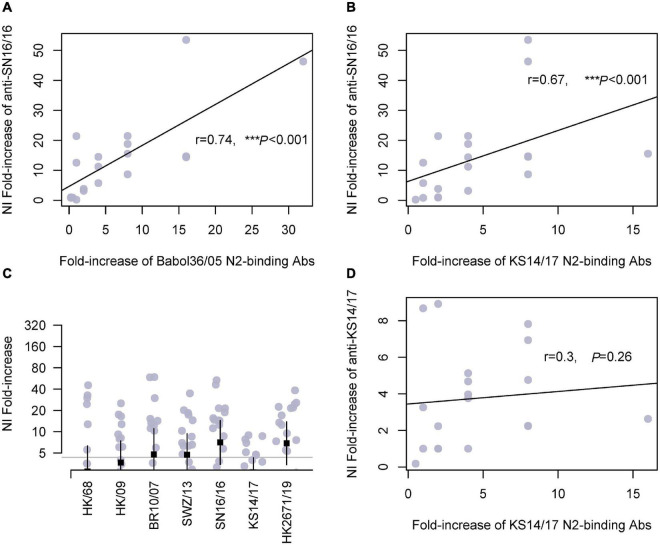
Cross-reactivity of serum N2-binding Abs in children and correlations with NI Abs. **(A)** Correlation of fold increase between anti-SN16/16 NI Ab and N2-binding Ab of A/Babol/36/05(H3N2, Babol36/05). **(B)** Correlation of fold increase between anti-SN16/16 NI Ab and N2-binding Ab of A/Kansas/14/2017(H3N2, KS14/17). **(C)** Fold increase of NI Abs against RG H6N2 viruses bearing N2 of A/Hongkong/1968(H3N2, HK/68), A/Hongkong/33982/2009(H9N2, HK/09), 2009H9N2(HK/09), A/Brisbane/10/2007(H3N2, Br10/07), A/Switzerland/9715293/2013(H3N2, SWZ/13), SN16/16, A/KS14/17, and Hongkong/2671/2019(H3N2, HK2671/19), respectively. **(D)** Correlations between anti-KS14/17 NI and KS14/17-binding Abs in children. The gray line **(C)** is 4, and the positive conversion is fold increase ≥ 4. The line in **(A,B,D)** is the regression line. The mean ± 95% CI is shown. The *r*- and *p*-values are indicated. *** < 0.001.

Herein, we tested the cross-reactivities of the sera to a serial of N2. We measured the NA-binding and NI Abs. Similar trends were found in children and adults (Figure 3 and Supplementary Figure 2, respectively). The antisera cross-bound with 2005N2(Babol36/05) and 2017N2(KS14/17) and cross-inhibited the N2 activity of 2007N2(Br10/2007) and 2013N2(SWZ/13), even the avian-origin N2 such as 1968N2 and 2009H9N2. We discovered a significant correlation between serum anti-SN16/16 MN Abs and KS14/17-binding Abs in children (*r* = 0.52, *p = 0.02; Supplementary Figure 3). Furthermore, the associations exist between anti-SN16/16 NI and Babol36/05-binding (*r* = 0.74, ***p < 0.001) or KS14/17-binding Abs (*r* = 0.67, ***p < 0.001) but not between anti-KS14/17 NI and KS14/17-binding Abs (*r* = 0.3, *P* = 0.26) (Figure 3). The discrepancy between anti-KS14/17 NI and N2-binding Abs suggests the conformational structure of NA enzyme was recognized by Abs in NI assay, which is a functional evaluation of their interactions. The information generally confirmed the contribution of anti-NA Abs to host protective immunity, and the wide spectrum of response to older NAs was related to the conserved property of NA. For instance, the D’197-199 was highly conserved, as shown in Supplementary Table 2. We speculated that the varied phenotype of NI against SN16/16 and KS14/17 is due to the genetic mutations mentioned previously.

### N-Glycosylation at 329 and K344E Recovering the Reduced NA Activity Inhibition Activity to the Recent Dominant H3N2 Virus

**FIGURE 4:**
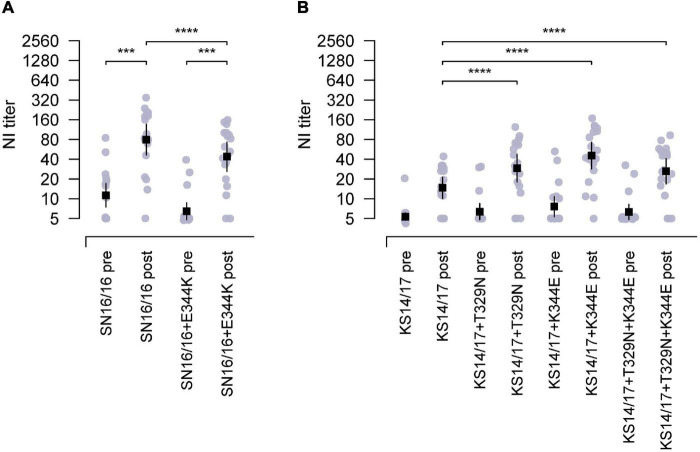
The epitopes of 329 *N*-glycosylation and E344 targeting by the antisera from children during 2018–2019. **(A)** Reduced NI activity of the antisera in children to E344K mutants using RGH6N1-SN16/16 + E344K compared with RGH6N1-SN16/16. **(B)** Enhanced NI activity of antisera in children to RGH6N1-KS14/17 + T329N, RGH6N1-KS14/17 + K344E, or RGH6N1-KS14/17 + T329N + K344E compared with RGH6N1-KS14/17. The sera of children collected in the pre– and post–flu season were for tests. The mean ± 95% CI is shown. *p*-value is indicated. *** < 0.001, **** < 0.0001.

Then, we proceeded with the *in vivo* testing using recombinant H6N2 variant (SN16/16 + E344K) with a single E344K mutation in SN16/16 N2. This variant had an epitope of *N*-glycosylation at 329 and E344K. The antisera from children’s cases displayed a significantly lower reactivity to the variant than SN16/16 (Figure 4A, ***p < 0.001). On the other hand, we generated N2 mutants with the KS14/17 NA backbone with one or two combinational mutations that had an epitope of *N*-glycosylation at 329 or E344 or both. Only a single mutation, 329 glycans or K344E, could upregulate the reactivation of children’s antisera to KS14/17, while the effects of the combinational mutations showed a moderate rise, implying a possible local fitness in the antigenic region (Figure 4B, ***p < 0.001). The NI activities of children antisera to KS17 variants were enhanced, although the NI titers did not reach the significantly comparable level as that to SN16/16 (Supplementary Table 4). We also discovered the children’s antisera demonstrated similar NI reactivity to HK2671/19 virus, bearing 329 non-glycans and K344E, as to SN16/16. Those differential NI activities suggest that other mechanisms might be involved. We also tested those variants using the ferret antisera challenged by the wild-type H3N2 viruses (Supplementary Table 3). The NA antigen ratio of SN16/16 and KS14/17 was greater than 1.5 and demonstrated antigenicity difference between the two NAs. Limited by the available volume of human sera, we performed the NI tests on SN16/16 and KS14/17 variants using ferret antisera. One-way interaction data suggest some differences among the NA variants compared with wild-type NA. Similar enhanced NI activities to KS14/17 variants bearing T329N and/or K344E were found. Of interest, the mutation N329T of SN16/16 also enhanced the NI activity of ferret sera and reversed the downregulation effects of E344K, implying complex interactions presented among antigenic sites and host Ab profile. The findings support that the amino acids in sites 329 and 344 are the key determinants for NA antigenicity.

## Discussion

The surface glycoproteins, HA and NA of the influenza virus, are the two major antigens triggering immune responses in the host (Schulman and Kilbourne, 1969). The global head of HA is highly variant, and NA is relatively conserved. For HI, the assay is conventionally used to characterize antigenicity, diagnosis, or vaccine evaluation (Schulman and Kilbourne, 1969; Eichelberger and Wan, 2015; Xu et al., 2021), while it is limited in the current seasonal H3N2 virus experiments. Anti-NA Abs could alleviate clinical severity by inhibiting viral release from infected cells (Schulman, 1969; Couch et al., 2013; Eichelberger and Wan, 2015). Therefore, we studied the anti-N2 response in the serologically confirmed H3N2 infection cases and expected its use for H3N2. Furthermore, the surging of 3C.3a H3N2 in middle-aged people in North America in the 2019/20 flu season drives us (Skowronski et al., 2019) to compare the Ab responses between adults and children.

Our work demonstrated that NI Ab seroconversion did occur in those individuals who seroconverted by neutralizing Abs against circulating 3C.2a1-like H3N2 virus in 2018/2019 flu season. The children’s cases show a stronger response manifesting as a higher NI titer and seroconversion rate than adults. Our data support the finding that NI Ab response is frequently observed in the affected children (Huang et al., 2019). In addition, the correlation of MN Abs and NI Abs and HA-binding in our studied age group of 7–17 years old suggests the involvement of anti-HA and anti-NA Abs in the protective immunity.

Conversely, the associations between serum MN Abs and SN16/16-NI Abs or H3-binding Abs in adults could not be detected. It is well studied that the levels of cross-reactive Abs against older strains accumulated along with increasing age or exposures, and the strain-specific Ab is relatively weaker (Fonville et al., 2014). Thus, an Ab landscape against a serial of HA or NA but not only the response to a specific strain might be appropriate for assessing adults’ responses.

The broad cross-reactivity and long-term persistence of NI Abs against 1957–2014 N2 of seasonal H3N2 in humans have been reported (Rajendran et al., 2017; Chen et al., 2018). The cross-reactivity is indeed observed in our study. The NI activities of the antisera target 2007 and 2013 seasonal N2 of different HA genetic clades of the H3N2 virus and the avian-origin N2 represented by the 1968 N2 and H9N2. In combination with NA-binding enzyme-linked immunosorbent assay, a conflict was found in tests using N2 of A/Kansas 14/17(H3N2, 3C.3a). This N2 belongs to the N2 of 3C.2a H3N2, whereas it bears non-*N*-glycosylation in 329 and K in 344, which is different from the earlier 3C.2a viruses of 2014. Of interest, this molecular signature usually occurred in the subsequent N2 of 3C.3a, finally retained in contemporary 3C.2a and 3C.3a H3N2 viruses. We identified their contributions to NA antigenicity drift by using mutants and reverse mutants, and the NI titer of the antisera to the virus with the epitopes of non-glycosylation in 329 and 344K markedly reduced, particularly to 344K.

To date, far less is known about N2 epitopes of anti-NA in humans than anti-HA (McAuley et al., 2019). The known epitopes such as G248 and G429 or some enzyme active sites are recognized by some NI-positive anti-N2 monoclonal Abs (Chen et al., 2018; Stadlbauer et al., 2019). Sites 329 and 344 in N2 are located in the antigenic sites F’ and G’, respectively (Fanning et al., 2000). Crystal structure studies have been demonstrated that site 329 is adjacent to 344, which is located at the edge of the active site pocket (Wu et al., 2019). The two sites are thought to compose the local net charge of the antigenic region on N2, and the opposite charge changes on some pairwise residues in the region likely to constrain the NA antigenicity (Wang et al., 2021). The glycan change at 329 and E344K substitution might balance the local charge net of the antigenic region. Furthermore, both sites are near the polar residues of 325–340, a highly flexible domain (Colman, 1994; Fanning et al., 2000; Wu et al., 2019; UniProtKB, 2021). Once being interrupted by Abs, the binding of NA to sialic acid substance might change. Herein, the prevalence of N2 with such alternative epitopes we reported and the high frequency of NA antigenicity drift including N1 and N2 (Sandbulte et al., 2011; Changsom et al., 2016; Gao et al., 2019; Wan et al., 2019) highlight the urgency to understand the mechanism.

Altogether, our findings suggest that NI assay could be applied in the serological diagnosis for H3N2 infection in children, and the antigenic drift of current N2 should be considered for vaccine selection.

## Data Availability Statement

The original contributions presented in the study are included in the article/Supplementary Material, further inquiries can be directed to the corresponding author/s.

## Ethics Statement

The studies involving human participants were reviewed and approved by the National Institute for Viral Disease Control and Prevention, China CDC. Written informed consent to participate in this study was provided by the participants’ legal guardian/next of kin.

## Author Contributions

JZ and CX designed the experiments. WH, LX, and DW provided the viral RNAs and suggestions. JG, XL, ZL, LQL, JL, JFG, CX, and JZ performed the experiments. LL and CX collected and transferred samples. JG, XL, JLG, KQ, CX, and JZ analyzed the data. JZ, JLG, and XL wrote the manuscript. JZ, XL, and KQ revised the manuscript. All authors contributed to the article and approved the submitted version.

## Author Disclaimer

The contents of this article are solely the authors’ responsibility and do not necessarily represent the views of China CDC and other organizations.

## Conflict of Interest

The authors declare that the research was conducted in the absence of any commercial or financial relationships that could be construed as a potential conflict of interest.

## Publisher’s Note

All claims expressed in this article are solely those of the authors and do not necessarily represent those of their affiliated organizations, or those of the publisher, the editors and the reviewers. Any product that may be evaluated in this article, or claim that may be made by its manufacturer, is not guaranteed or endorsed by the publisher.

## Footnotes

1
http://www.R-project.org
