# Supplementary material for: The Antibody Response Against Neuraminidase in Human Influenza A (H3N2) Virus Infections During 2018/2019 Flu Season: Focusing on the Epitopes of 329-N-Glycosylation and E344 in N2
Source: Front Microbiol. 2022 Mar 21;13:845088. doi: 10.3389/fmicb.2022.845088 (PMC8978628; doi:10.3389/fmicb.2022.845088)
Supplement: Supplementary file 1 [file Data_Sheet_1.docx]

Supplementary Figure S1. Phylogenetic tree for subtype N2 influenza A viruses based on the

representative neuraminidase protein sequences from 1968 to 2021.


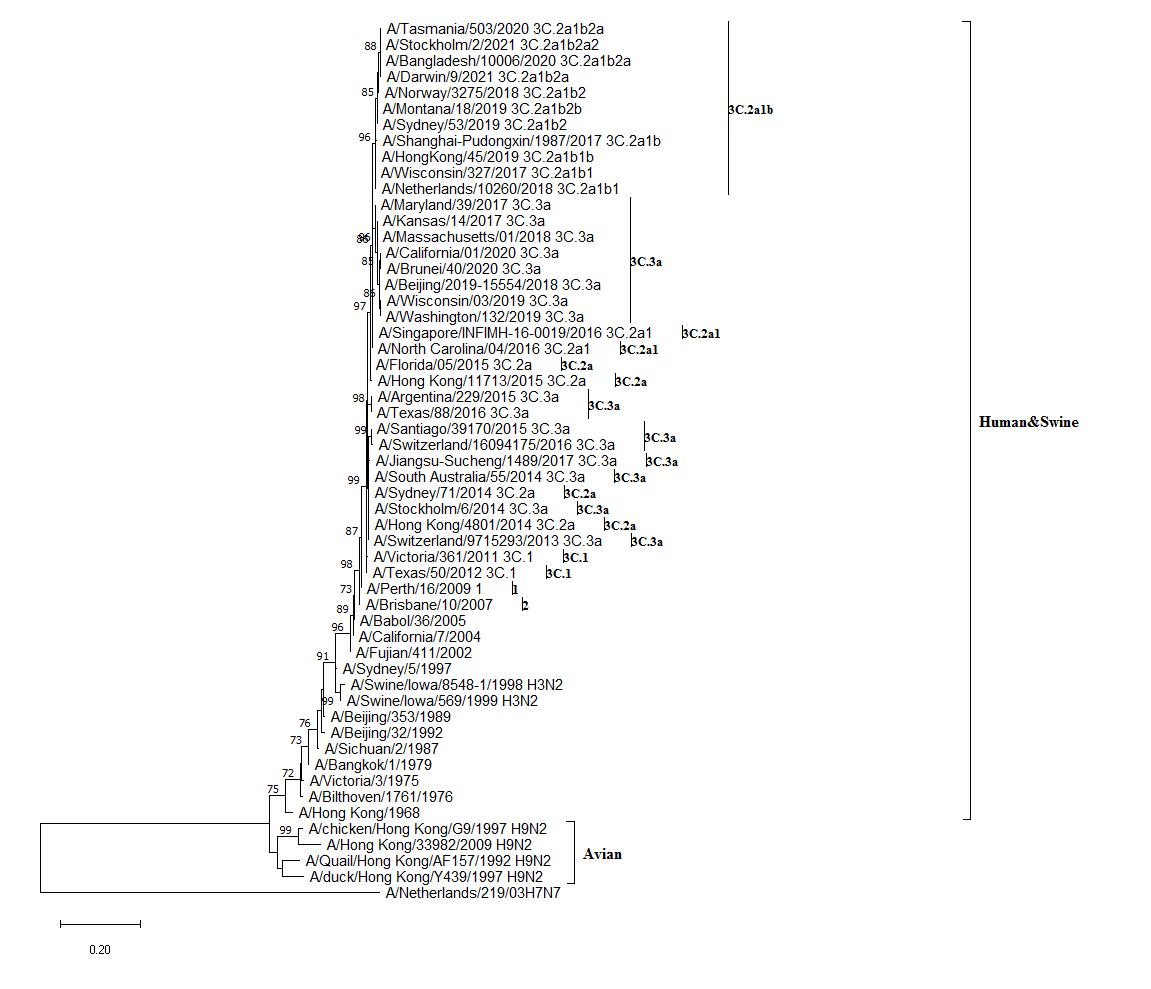
The full-length neuraminidase protein sequences were downloaded from the Global Initiative on Sharing All Influenza Data database (GISAID) and the NCBI (Influenza Virus Resource). Bioedit 7 was used for alignment and analysis of amino acid residues. The phylogenetic tree for neuraminidase protein sequences of selected influenza viruses was constructed with MEGA X. The Maximum Likelihood method was used for phylogenetic analysis with the JTT+G+I model. The tree topology was evaluated by 1000 bootstrap replicates and only bootstraps greater than 70% were shown at each node. A/Netherlands/219/2003(H7N7) was chosen as outgroup.
