# Supplementary material for: The Antibody Response Against Neuraminidase in Human Influenza A (H3N2) Virus Infections During 2018/2019 Flu Season: Focusing on the Epitopes of 329-N-Glycosylation and E344 in N2
Source: Front Microbiol. 2022 Mar 21;13:845088. doi: 10.3389/fmicb.2022.845088 (PMC8978628; doi:10.3389/fmicb.2022.845088)

Supplementary Figure S2. Cross-reactivity of serum N2-binding Abs in adults and correlations with NI Abs. (A) the correlation of anti-SN16/16 between N2-binding Abs against Barbol36/05; (B) the correlation of anti-SN16/16 between N2-binding Abs against KS14/17;(C) the Fold-increase of NI Abs against RG H6N2 viruses bearing N2 of 1968H3N2(HK/68), 2009H9N2(HK/09), Br10/07, SWZ/13, SN16/16, KS14/17 and HK2671/19; (D) the correlations between anti-KS14/17 NI and KS14/17-binding Abs in adults. The line in(A), (B) and (D) is the regression line. The mean ± 95% CI is shown. The r and *P*-values are indicated. *<0.05.


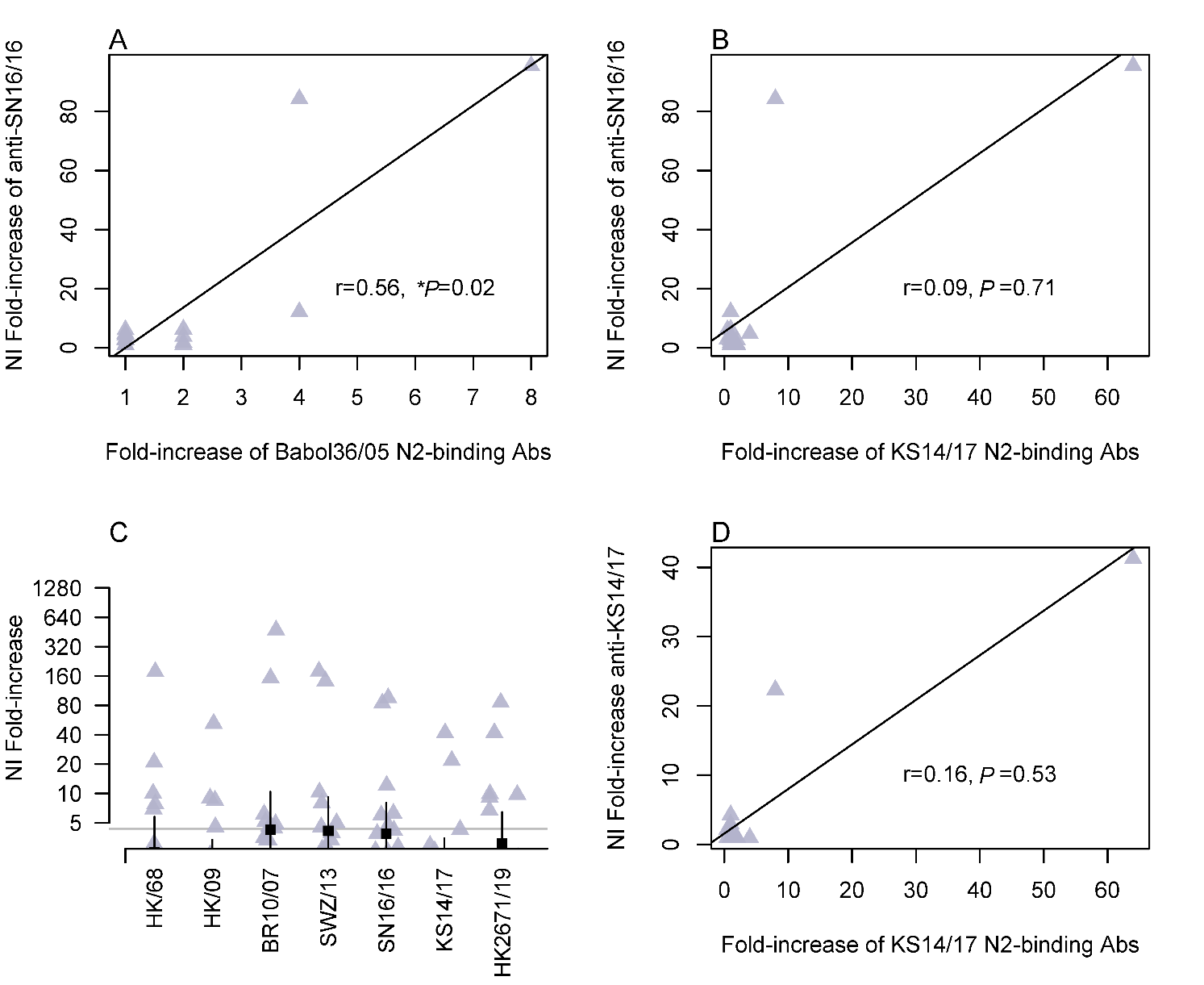

Supplement: Supplementary file 2 [file Data_Sheet_2.docx]
