# Supplementary material for: The Antibody Response Against Neuraminidase in Human Influenza A (H3N2) Virus Infections During 2018/2019 Flu Season: Focusing on the Epitopes of 329-N-Glycosylation and E344 in N2
Source: Front Microbiol. 2022 Mar 21;13:845088. doi: 10.3389/fmicb.2022.845088 (PMC8978628; doi:10.3389/fmicb.2022.845088)

Supplementary Figure S3. Correlation of MN Abs and the KS14/17 N2-binding Abs. (A) the correlation of anti-SN16/16 MN Abs between N2-binding Abs against KS14/17 in children; (B) the correlation of anti-SN16/16 MN Abs between N2-binding Abs against KS14/17 in adults; The line in(A) and (B) is the regression line. The r and *P*-values are indicated. *<0.05.


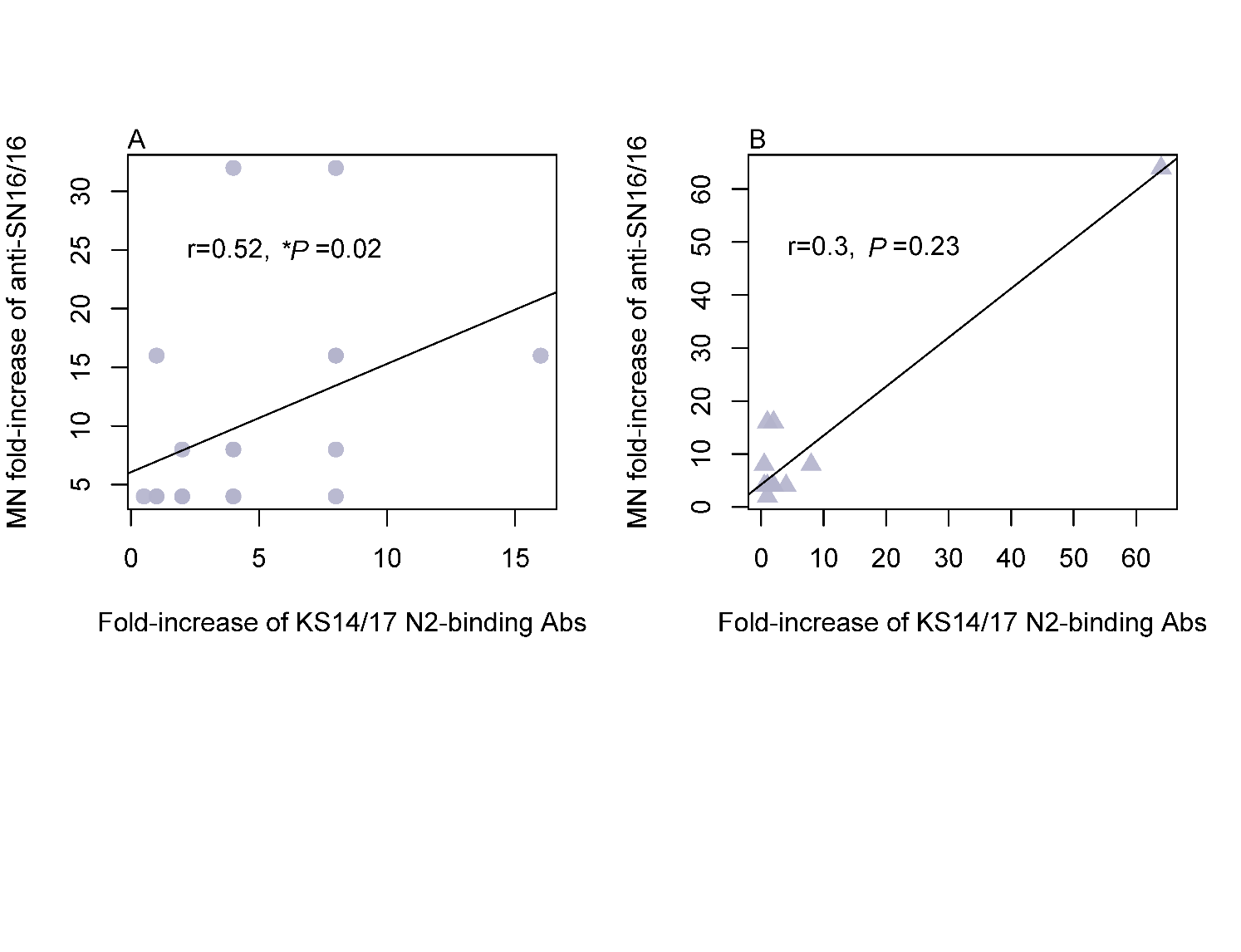

Supplement: Supplementary file 3 [file Data_Sheet_3.docx]
