# Supplementary material for: The Antibody Response Against Neuraminidase in Human Influenza A (H3N2) Virus Infections During 2018/2019 Flu Season: Focusing on the Epitopes of 329-N-Glycosylation and E344 in N2
Source: Front Microbiol. 2022 Mar 21;13:845088. doi: 10.3389/fmicb.2022.845088 (PMC8978628; doi:10.3389/fmicb.2022.845088)
Supplement: Supplementary file 5 [file Table_1.docx]

Table S1. Information of the samples in this study.

| **Patient ID** | **Age** | **Gender** | **MN titer** | | **HI titer** | | | | | | | | **H1 positive** |
| --- | --- | --- | --- | --- | --- | --- | --- | --- | --- | --- | --- | --- | --- |
|  |  |  | **Pre- (SN16/16)** | **Post-  (SN16/16)** | **Pre-  H1** | **Post-  H1** | **Pre-  H3** | **Post-  H3** | **Pre- BY** | **Post-  BY** | **Pre-  BV** | **Post-  BV** |  |
| A009 | 60 | F | 20 | 80 | 5 | 5 | 320 | 80 | 10 | 5 | 5 | 5 |  |
| A015 | 45 | F | 5 | 20 | 40 | 40 | 5 | 40 | 20 | 20 | 5 | 5 |  |
| A041 | 44 | F | 5 | 80 | 20 | 80 | 40 | 40 | 5 | 20 | 5 | 20 | Y |
| A047 | 39 | F | 20 | 80 | 10 | 10 | 40 | 160 | 40 | 40 | 5 | 5 |  |
| A074 | 41 | F | 5 | 20 | 10 | 10 | 10 | 20 | 40 | 40 | 20 | 20 |  |
| A092 | 44 | M | 20 | 80 | 10 | 10 | 80 | 160 | 80 | 5 | 40 | 20 |  |
| A144 | 33 | F | 80 | 160 | 10 | 80 | 40 | 80 | 80 | 80 | 5 | 5 | Y |
| A163 | 21 | M | 40 | 160 | 320 | 320 | 40 | 320 | 80 | 80 | 5 | 5 |  |
| A168 | 36 | F | 20 | 160 | 40 | 80 | 20 | 640 | 5 | 40 | 20 | 5 |  |
| A171 | 38 | F | 10 | 640 | 5 | 40 | 20 | 640 | 5 | 40 | 5 | 5 | Y |
| A179 | 25 | F | 5 | 20 | 40 | 40 | 40 | 40 | 40 | 320 | 5 | 5 |  |
| A277 | 38 | F | 10 | 40 | 10 | 10 | 10 | 160 | 5 | 5 | 5 | 5 |  |
| A401 | 34 | F | 10 | 80 | 5 | 5 | 5 | 160 | 40 | 20 | 5 | 5 |  |
| A435 | 47 | M | 10 | 160 | 10 | 40 | 10 | 80 | 5 | 5 | 5 | 5 | Y |
| A447 | 51 | M | 10 | 40 | 5 | 40 | 20 | 20 | 5 | 5 | 5 | 5 | Y |
| A473 | 56 | M | 10 | 40 | 10 | 10 | 20 | 40 | 20 | 20 | 5 | 5 |  |
| A478 | 44 | F | 10 | 40 | 5 | 5 | 20 | 40 | 10 | 5 | 10 | 10 |  |
| C001 | 9 | F | 20 | 80 | 160 | 80 | 80 | 160 | 160 | 80 | 40 | 20 |  |
| C011 | 8 | M | 20 | 80 | 40 | 1280 | 80 | 80 | 80 | 160 | 5 | 10 | Y |
| C013 | 7 | M | 10 | 160 | 5 | 5 | 10 | 160 | 10 | 5 | 5 | 5 |  |
| C015 | 8 | M | 5 | 160 | 80 | 80 | 20 | 320 | 10 | 20 | 5 | 5 |  |
| C020 | 7 | F | 5 | 160 | 40 | 80 | 5 | 320 | 160 | 160 | 5 | 5 |  |
| C045 | 11 | M | 20 | 80 | 160 | 80 | 20 | 40 | 80 | 5 | 5 | 5 |  |
| C052 | 9 | F | 20 | 80 | 80 | 80 | 40 | 320 | 80 | 80 | 5 | 5 |  |
| C080 | 12 | F | 10 | 40 | 20 | 20 | 20 | 80 | 160 | 10 | 10 | 10 |  |
| C084 | 9 | M | 5 | 80 | 40 | 40 | 40 | 160 | 80 | 40 | 5 | 5 |  |
| C135 | 9 | F | 20 | 80 | 320 | 320 | 20 | 160 | 80 | 40 | 5 | 5 |  |
| C140 | 8 | M | 10 | 40 | 80 | 80 | 160 | 640 | 40 | 40 | 5 | 5 |  |
| C163 | 11 | M | 10 | 160 | 20 | 20 | 20 | 640 | 80 | 80 | 10 | 5 |  |
| C180 | 9 | F | 40 | 160 | 640 | 320 | 160 | 640 | 320 | 160 | 5 | 5 |  |
| C184 | 9 | M | 40 | 320 | 40 | 160 | 80 | 320 | 40 | 40 | 5 | 5 | Y |
| C201 | 9 | M | 40 | 320 | 10 | 40 | 20 | 160 | 80 | 80 | 5 | 5 | Y |
| C251 | 10 | F | 20 | 320 | 40 | 40 | 40 | 640 | 80 | 80 | 160 | 80 |  |
| C295 | 7 | M | 20 | 160 | 40 | 40 | 20 | 320 | 80 | 160 | 5 | 5 |  |
| C365 | 17 | F | 10 | 80 | 40 | 40 | 40 | 40 | 40 | 40 | 10 | 10 |  |
| C372 | 17 | F | 10 | 40 | 5 | 5 | 20 | 40 | 5 | 5 | 5 | 5 |  |

BY, B/ Phuket/3073/2013 (B Yamagata lineage); BV, B/Colorado/06/2017 (B Victoria lineage); A/H1N1, A/Michigan/45/2015 (H1N1) pdm09; A/H3N2, A/Singapore/INFIMH-16-0019/2016(H3N2); H1 positive, H1 HI titer fold ≥ 4. 　 denotes no. Y denotes yes.
