# Supplementary material for: The Antibody Response Against Neuraminidase in Human Influenza A (H3N2) Virus Infections During 2018/2019 Flu Season: Focusing on the Epitopes of 329-N-Glycosylation and E344 in N2
Source: Front Microbiol. 2022 Mar 21;13:845088. doi: 10.3389/fmicb.2022.845088 (PMC8978628; doi:10.3389/fmicb.2022.845088)
Supplement: Supplementary file 6 [file Table_2.docx]

Table S2. The amino acid substitution at the antigenic epitopes in the head of N2, including D’197-199, F’329-339, G’ 344-347, I’ 368-369 and K’ 400-403 as well as the regions of E’302-308, H’356-358, J’384-386 and L’463-468 of 1968-2021 representative H3N2 viruses.

| **HA Clade** | **NA sequence** | **The flu season for the vaccine strain in the northern hemisphere** | **D’**  **197-199** | | | **E’**  **302-308** | | | | | | | **F’**  **329-339** | | **G’**  **344-347** | | **H’**  **356-358** |  | **I’**  **368-369** |  | **J’**  **384-386** |  | **K’**  **400-403** | **L’**  **463-468** | | | |
| --- | --- | --- | --- | --- | --- | --- | --- | --- | --- | --- | --- | --- | --- | --- | --- | --- | --- | --- | --- | --- | --- | --- | --- | --- | --- | --- | --- |
|  |  |  | 197 | 198 | 199 | 302 | 303 | 304 | 305 | 306 | 307 | 308 | 329 | 339 | 344 | 346 | 356 | 367 | 369 | 380 | 385 | 392 | 402 | 463 | 464 | 465 | 468 |
| **-** | **A/Hongkong/1968 (H3N2)** | **-** | **D** | **D** | **K** | **V** | **V** | **D** | **I** | **N** | **M** | **E** | **D** | **N** | **R** | **N** | **S** | **S** | **D** | **I** | **T** | **I** | **N** | **N** | **I** | **N** | **P** |
| **-** | **A/Victoria/3/1975 (H3N2)** | **75/76** | **D** | **D** | **K** | **V** | **V** | **D** | **I** | **N** | **V** | **K** | **N** | **N** | **E** | **N** | **D** | **S** | **D** | **I** | **T** | **I** | **N** | **D** | **I** | **N** | **P** |
| **-** | **A/England/321/1977 (H3N2)** | **77/78** | **D** | **D** | **K** | **V** | **V** | **D** | **I** | **N** | **V** | **K** | **N** | **N** | **E** | **N** | **D** | **S** | **E** | **I** | **T** | **I** | **N** | **D** | **I** | **N** | **P** |
| **-** | **A/Bangkok/1/1979 (H3N2)** | **78/79&79/80** | **Y** | **D** | **K** | **V** | **V** | **D** | **I** | **N** | **V** | **K** | **N** | **N** | **E** | **N** | **D** | **G** | **E** | **I** | **T** | **I** | **N** | **D** | **I** | **N** | **P** |
| **-** | **A/Sichuan/2/1987 (H3N2)** | **88/89** | **H** | **D** | **E** | **I** | **V** | **D** | **I** | **N** | **V** | **K** | **N** | **N** | **E** | **S** | **D** | **G** | **E** | **I** | **T** | **I** | **N** | **D** | **I** | **N** | **P** |
| **-** | **A/Beijing/353/1987 (H3N2)** | **92-93** | **H** | **D** | **E** | **I** | **V** | **D** | **I** | **N** | **V** | **K** | **N** | **N** | **E** | **S** | **D** | **G** | **E** | **I** | **T** | **I** | **N** | **D** | **I** | **N** | **P** |
| **-** | **A/Beijing/32/1992 (H3N2)** | **93/94** | **H** | **D** | **E** | **I** | **V** | **D** | **I** | **N** | **V** | **K** | **N** | **N** | **E** | **S** | **D** | **S** | **K** | **I** | **T** | **I** | **N** | **D** | **I** | **N** | **P** |
| **-** | **A/Sydney/5/97 (H3N2)** | **98/99&99/00** | **H** | **D** | **E** | **I** | **V** | **D** | **I** | **N** | **V** | **K** | **N** | **N** | **E** | **G** | **D** | **S** | **K** | **I** | **K** | **I** | **N** | **D** | **I** | **N** | **P** |
| **-** | **A/Fujian/411/2002 (H3N2)** | **04/05** | **D** | **D** | **E** | **I** | **V** | **D** | **I** | **N** | **I** | **K** | **N** | **D** | **E** | **G** | **D** | **S** | **K** | **I** | **N** | **I** | **N** | **D** | **I** | **N** | **P** |
| **-** | **A/California/7/2004 (H3N2)** | **05/06** | **D** | **D** | **K** | **I** | **V** | **D** | **I** | **N** | **I** | **K** | **N** | **D** | **E** | **G** | **D** | **S** | **K** | **I** | **N** | **I** | **N** | **D** | **I** | **N** | **P** |
| **-** | **A/Babol/36/2005 (H3N2)** | **-** | **D** | **D** | **K** | **I** | **V** | **D** | **I** | **N** | **I** | **K** | **N** | **D** | **E** | **G** | **D** | **S** | **K** | **I** | **N** | **I** | **N** | **D** | **I** | **N** | **P** |
| **2** | **A/Brisbane/10/07 (H3N2)** | **08/09&09/10** | **D** | **D** | **K** | **I** | **V** | **D** | **I** | **N** | **I** | **K** | **N** | **D** | **E** | **G** | **D** | **N** | **K** | **I** | **N** | **I** | **N** | **D** | **I** | **N** | **P** |
| **1** | **A/Perth/16/2009 (H3N2)** | **10/11&11/12** | **D** | **D** | **K** | **I** | **V** | **D** | **I** | **N** | **I** | **K** | **N** | **D** | **E** | **G** | **D** | **N** | **K** | **I** | **N** | **I** | **N** | **D** | **I** | **N** | **P** |
| **3C.1** | **A/Victoria/361/2011 (H3N2)** | **12/13** | **D** | **D** | **K** | **I** | **V** | **D** | **I** | **N** | **I** | **K** | **N** | **D** | **E** | **G** | **D** | **N** | **T** | **I** | **N** | **I** | **D** | **D** | **L** | **N** | **P** |
| **3C.1** | **A/Texas/50/2012 (H3N2)** | **13/14&14/15** | **D** | **D** | **K** | **I** | **V** | **D** | **I** | **N** | **I** | **K** | **N** | **D** | **E** | **G** | **D** | **N** | **T** | **I** | **N** | **I** | **D** | **D** | **L** | **N** | **P** |
| **3C.3a** | **A/Switzerland/9715293/2013 (H3N2)** | **15/16** | **D** | **D** | **K** | **I** | **V** | **D** | **I** | **N** | **I** | **K** | **N** | **D** | **E** | **G** | **D** | **N** | **T** | **I** | **N** | **T** | **D** | **D** | **L** | **N** | **P** |
| **3C.2a** | **A/Hongkong/4801/2014 (H3N2)** | **16/17&17/18** | **D** | **D** | **K** | **I** | **V** | **D** | **I** | **N** | **I** | **K** | **N** | **D** | **E** | **G** | **D** | **N** | **T** | **I** | **N** | **T** | **D** | **D** | **L** | **N** | **P** |
| **3C.2a1** | **A/Singapore/INFIMH-16-0019/2016 (H3N2)** | **18/19** | **D** | **D** | **K** | **I** | **V** | **D** | **I** | **N** | **I** | **K** | **N** | **N** | **E** | **G** | **D** | **N** | **T** | **V** | **N** | **I** | **D** | **D** | **L** | **N** | **H** |
| **3C.3a** | **A/Kansas/14/2017 (H3N2)** | **19/20** | **D** | **D** | **K** | **I** | **V** | **D** | **I** | **N** | **I** | **K** | **T** | **N** | **K** | **G** | **D** | **N** | **T** | **V** | **N** | **I** | **D** | **D** | **L** | **N** | **H** |
| **3C.2a1b1b** | **A/Hongkong/2671/2019 (H3N2)** | **20/21** | **D** | **D** | **K** | **I** | **I** | **D** | **I** | **N** | **I** | **K** | **S** | **N** | **E** | **G** | **D** | **N** | **T** | **V** | **N** | **I** | **D** | **D** | **L** | **S** | **H** |
| **3C.2a1b2a** | **A/Darwin/9/2021 (H3N2)** | **-** | **D** | **D** | **K** | **I** | **I** | **D** | **I** | **N** | **I** | **K** | **S** | **N** | **K** | **D** | **D** | **N** | **T** | **V** | **N** | **I** | **D** | **N** | **I** | **S** | **H** |
| **-** | **A/Hongkong/33982/2009(H9N2)** | **-** | **D** | **D** | **K** | **V** | **L** | **F** | **I** | **N** | **M** | **A** | **D** | **D** | **R** | **A** | **S** | **S** | **D** | **D** | **T** | **I** | **N** | **N** | **I** | **N** | **p** |

- denotes no. The antigenic epitope region is highlighted with color.

|  |
| --- |
|  |
|  |
