# Supplementary material for: The Antibody Response Against Neuraminidase in Human Influenza A (H3N2) Virus Infections During 2018/2019 Flu Season: Focusing on the Epitopes of 329-N-Glycosylation and E344 in N2
Source: Front Microbiol. 2022 Mar 21;13:845088. doi: 10.3389/fmicb.2022.845088 (PMC8978628; doi:10.3389/fmicb.2022.845088)
Supplement: Supplementary file 7 [file Table_3.docx]

Table S3. NA antigenic analysis by using ferret antisera raised to wild-type viruses, SN/16/16(H3N2), KS/14/17(H3N2) and the antigenicity-like viruses.

| Ferret Antisera  Antigens | SN16/16(H3N2) | SN16/16(H3N2)-like | KS14/17(H3N2) | KS14/17(H3N2)-like |
| --- | --- | --- | --- | --- |
| RGH6N2(SN16/16) | 227.23 | 151.80 | 176.80 | 118.44 |
| RGH6N2(KS14/17) | 89.95 | 64.62 | 174.49 | 143.01 |
| Test variants |  |  |  |  |
| RGH6N2(SN16/16) +N329T | 287.42 | 198.23 | 264.84 | 173.4 |
| RGH6N2(SN16/16) +E344K | 105.20 | 76.85 | 84.62 | 58.57 |
| RGH6N2(SN16/16)+N329T+E344K | 200.02 | 159.56 | 356.81 | 412.71 |
| RGH6N2(KS14/17) +T329N | 124.24 | 86.40 | 127.20 | 83.23 |
| RGH6N2(KS14/17) +K344E | 164.05 | 125.19 | 234.59 | 128.71 |
| RGH6N2(KS14/17)+T329N+K344E | 108.53 | 72.30 | 117.46 | 69.17 |

SN16/16(H3N2)-like virus is A/Guizhou-Qingzhen/1968/2016 (H3N2, 3C.2a1); KS14/17(H3N2)-like virus is A/Beijing/2019-15554/2018 (H3N2, 3C.3a).
